# Stereotactic body radiotherapy for lung oligometastatic prostate cancer: An international retrospective multicenter study

**DOI:** 10.1016/j.ctro.2025.100944

**Published:** 2025-03-10

**Authors:** Maximilien Rogé, Patrick Bowden, Paul Conway, Ciro Franzese, Marta Scorsetti, Emmanuel Seront, Pierre Blanchard, Mario Terlizzi, Jonathan Khalifa, Corentin Pasquier, Ulrike Shick, Shankar Siva, Julie Paul, Stéphane Supiot

**Affiliations:** a Department of Radiation Oncology, Henri Becquerel Cancer Institute, 76000 Rouen, France; b Institut de Cancérologie de l’Ouest, F-44805 Saint Herblain, France; c Department of Radiation Oncology, Icon Cancer Centre, Richmond, Victoria, Australia; d Department of Radiation Oncology, IRCCS Humanitas Research Hospital, Milano, Italy; e Department of Medical Oncology, Institut Roi Albert II, Cliniques Universitaires Saint Luc, Brussel, Belgium; f Department of Radiation Oncology, Institut Gustave Roussy, Villejuif, France; g Department of Radiation Oncology, Institut Universitaire du Cancer de Toulouse, Toulouse, France; h Department of Radiation Oncology, University Hospital Morvan, 2 avenue Foch, 29200 Brest, France; i Sir Peter MacCallum Department of Oncology, Peter MacCallum Cancer Centre, University of Melbourne, Australia

**Keywords:** Androgen deprivation therapy, Biochemical progression free survival, Lung metastases, Prostate cancer, Oligometastases, Stereotactic body radiotherapy

## Abstract

**Background:**

Management of prostate cancer (PCa) patients with lung oligometastases remains unclear in the absence of published data.

**Objective:**

The aim of this study was to evaluate the efficacy of Stereotactic Body Radiotherapy (SBRT) in this setting.

**Design, setting, and participants:**

We conducted a multicenter retrospective study that included 35 PCa patients treated with SBRT for lung oligometastases in 7 centers across 3 countries.

**Outcome measurements and statistical analysis:**

The efficacy was evaluated by the progression free-survival (PFS), defined as pre-SBRT PSA + 25 % or nadir PSA + 25 % and + 2 ng/mL or radiological progression if it occurred before biochemical progression. The local recurrence free-survival (LRFS), distant metastases free-survival (DMFS), Overall Survival (OS) and Androgen Deprivation Therapy free-survival were also assessed. Survival was estimated using the Kaplan Meier method.

**Results:**

35 patients were included with lung oligometastases staged with PET-CT for 97 % and histologically biopsy confirmed for 51 %. 77 % had an oligorecurrent metastatic disease. The median pre SBRT PSA was at 1.7 ng/mL [0.8, 3.0] and the median follow-up after SBRT was 28.7 months. The median PFS was 21.6 months [95 %CI: 21.6; NA] and the median DMFS was 32.4 months [95 %CI: 22.2–NA]. No parameters were significantly associated with PFS on the univariate and multivariate models.

For patients who did not start ADT before or concomitantly with SBRT (n = 18), the 1-year ADT free-survival rate was estimated at 87.2 % [71.9;100].

**Conclusions:**

SBRT for PCa lung oligometastases offers good oncological outcomes, comparable to those reported for bone and/or lymph node metastases SBRT. Our results encourage the inclusion of patients with lung oligometastatic disease in clinical trials designed to assess the value of SBRT.

**Patient summary:**

SBRT for prostate cancer lung oligometastases shows promising results, similar to treatments for bone or lymph node oligometastases.

## Highlights

• SBRT for lung oligometastases in PCa has demonstrated excellent local control.
• This approach offers good biochemical control.
• Outcomes are comparable to those reported for bone and/or lymph node metastases.
• These findings support the inclusion of such patients in clinical trials.

## Introduction

The management of metastatic prostate cancer (PCa) has undergone significant advancements over the past decades, driven by progress in systemic treatments such as chemotherapy and next-generation hormone therapies [[1], [2], [3], [4], [5]].

Alongside these developments, radiotherapy has assumed a pivotal role at various stages of metastatic disease.

In patients with de novo low volume metastatic prostate cancer, prostate radiotherapy has been shown to improve overall survival, as demonstrated in the STAMPEDE trial [6]. More recently, the PEACE1 trial highlighted that prostate radiotherapy also improves radiographic progression-free survival and castration-resistant-free survival while reducing the incidence of serious genitourinary events [7].

In cases of metachronous oligometastatic prostate cancer, the potential benefits of integrating local metastasis-directed therapy have been explored in three dedicated randomized phase 2 studies: STOMP, ORIOLE, and EXTENT [[8], [9], [10]]. These trials provided encouraging evidence supporting the use of SBRT in this setting. Nevertheless, due to the limited number of patients with visceral metastases included, these results cannot be directly applied to this subpopulation.

In metastatic PCa, several studies have demonstrated the poorer prognosis of visceral metastases compared to bone or lymph node metastases, suggesting that local treatment may be less beneficial in this subpopulation due to the higher risk of disease progression [11]. It has also been suggested that visceral metastatic PCa is not a uniform stage, with lung metastases being associated with a better prognosis compared to other types of visceral metastases [[12], [13], [14]]. Despite this observation, there are no specific retrospective or prospective data on the outcomes of PCa patients with lung oligometastases treated with Stereotactic Body Radiotherapy (SBRT).

We report the largest multicenter retrospective series of SBRT of lung oligometastases in PCa patients. The aim of this study is to evaluate the efficacy and toxicity of SBRT in this setting.

## Material and methods

### Inclusion/exclusion criteria

All consecutive patients who met the eligibility criteria in the participating centers were retrospectively included in the study.

The inclusion criteria were as follows: men who had histologically confirmed PCa and who experienced synchronous or metachronous oligometastatic disease with 5 or less metastases with at least one lung metastasis.

All the metastases must be treated with local treatment with or without systemic treatment. Patients could be treated with a multimodality approach including surgery and/or interventional radiology but at least one lung metastasis should be treated with SBRT. Biopsy was not mandatory if all diagnostic elements were univocal.

### Treatments received

As the study was retrospective, there was no common scheme SBRT protocol. However, the 2 Gy equivalent dose was calculated using an alpha/beta ratio of 3 Gy for prostate cancer cells. We also reported the PTV volume in cubic centimeter (cc).

### Endpoints

The primary objective was to evaluate the efficacy of SBRT in terms of disease control. Progression-free survival (PFS) was defined at the time from SBRT to first PSA reaching a value that is ≥ 25 % from pre-SBRT PSA or first PSA reaching a value that is ≥ 25 % and + 2 ng/mL from PSA nadir or a radiological local or distant progression if it occurred before biochemical progression.

Secondary objectives included local control, distant progression, ADT initiation, overall survival (OS) and toxicities (according to CTCAE v5). Local progression was defined as progression of a treated metastasis while distant progression was defined by the appearance of new metastatic lesion (PET or CT scan). Androgen Deprivation Therapy (ADT)-free survival was defined for ADT-naive patients from the end of SBRT to ADT initiation.

Data were censored at the date of the last news for patients who were still alive and did not relapse.

### Statistical analysis

Categorical variables were described using the number of people and its associated percentage. Quantitative variables were described using median and interquartile range (Q1-Q3). All survival times were calculated from the end of SBRT and estimated by the Kaplan-Meier method with 95 % confidence intervals (CI). Patients who did not experience the event of interest were censored at their last follow-up. For independent prognostic factors for event occurrence over time, univariable analyses were performed using Cox proportional hazards models where the proportional hazards hypothesis was tested. Variables significant at the 20 % level in univariable were entered into a multivariable model with a final significance level set at 5 % (two-sided formulation). The strength of the association was estimated by the adjusted hazard ratio (HR), reported with a 95 % CI. Missing data were described for each variable, but no imputation was performed. All analyses were performed with R software [R Core Team (2014). R: A language and environment for statistical computing. R Foundation for Statistical Computing, Vienna, Austria. URL: https://www.R-project.org/.].

### Data collection and regulatory aspects

In accordance with French regulations, the ICO is committed to following the MR-004 reference methodology, under number 415. The protocol was approved by the Ethics Committee of Angers University (number 2021–219).

Every participating center was responsible for ensuring that patients did not object to the use of their clinical data for research purposes and that it complied with the specific legislation of their country.

## Results

### Population

We included 35 patients from 7 centers across 3 countries (Table S1) who underwent SBRT for lung oligometastatic disease between April 2014 and August 2022, with half of patients treated after January 2020. The primary tumor characteristics are shown in Table 1.

**Table 1:** Primary tumor characteristics.

	**Total** **(N = 35)**
**Age at PCa diagnosis (years)**	
Median [Q1, Q3]	64 [57, 69]
**Initial PSA at diagnosis (ng/mL)**	
Median [Q1, Q3]	7.8 [6.1, 12.5]
Missing	9
**ISUP**	
1	2 (6 %)
2	10 (30 %)
3	9 (27 %)
4	6 (18 %)
5	6 (18 %)
Missing	2
**Prostate treatment**	
Radical prostatectomy alone	6 (17 %)
Radical prostatectomy and Radiotherapy	23 (66 %)
Radiotherapy alone	4 (11 %)
No local treatment	2 (6 %)

Footnote: PCa: Prostate Cancer; ISUP: International Society of Urological Pathology.

The median age at PCa diagnosis was 64 (57–69) years, with a median initial PSA at 7.8 (6.1–12.5) ng/mL. The majority of patients (n = 33, 94.3 %) underwent local prostate treatment with 23 patients (66 %) treated with prostatectomy followed by salvage radiotherapy.

### Metastatic disease

The characteristics of metastatic disease are shown in Table 2.

**Table 2:** Metastatic disease characteristics.

	Total
	(N = 35)
Castration status at the metastatic diagnosis	
mCRPC	1 (3 %)
mCSPC	34 (97 %)
**Classification of Lung Oligometastatic Disease**	
Synchronous oligometastatic disease	2 (6 %)
Metachronous oligorecurrent disease	27 (77 %)
Oligoprogressive disease	3 (9 %)
Oligopersistent disease	3 (9 %)
**Number of Lung metastases**	
1	22 (63 %)
2	10 (29 %)
3	3 (9 %)
**Metastatic imaging workup**	
CT	1 (3 %)
PET-CT Choline	16 (46 %)
PET-CT PSMA	18 (51 %)
**Biopsy-proven lung metastasis**	
No	17 (49 %)
Yes	18 (51 %)

Footnote: mCRPC: Metastatic Castration Resistant Prostate Cancer; mCSPC: Metastatic Castration Sensitive Prostate Cancer. PET: Positron Emission Tomography. CT: Computed Tomography.

The majority of patients (n = 34, 97 %) were castration-sensitive at the time of first metastatic diagnosis. Thirty-two patients (91 %) had two or fewer lung metastasis at the metastatic disease diagnosis. 27 patients (77 %) had a metachronous oligorecurrent oligometastatic disease. A PET-CT Choline (n = 16, 46 %) or PSMA (n = 18, 51 %) was performed on 34 patients (97 %). The only patient without PET-CT had a biopsy proven lung metastasis. About one half of patients had a biopsy-proven lung metastasis (n = 18, 51 %).

### Stereotactic body radiotherapy

Details of the patients' characteristics at the time of SBRT, as well as the specifics of the SBRT treatment, are provided in Table 3. The median time between lung metastasis diagnosis and SBRT was 4 months, with a median pre-SBRT PSA level of 1.7 ng/mL. Most patients (77 %) had a single lung metastasis treated with SBRT, while 20 % had two metastases, and 3 % had three metastases. Five patients underwent multimodal therapy for the management of their lung oligometastases. Among them, three patients, each diagnosed with two pulmonary metastases, had one metastasis surgically removed and the other treated with SBRT. Two patients with three pulmonary metastases had two of the metastases treated with radiofrequency ablation, followed by SBRT for the remaining lesion. The median planning target volume (PTV) was 21.5 cc, and the median equivalent dose in 2 Gy fractions (EQD2Gy) was 130 Gy. Seventeen patients (49 %) started ADT before or concomitantly with SBRT with a median duration of 16 months.

**Table 3:** Stereotactic Body Radiotherapy Details.

	**Total**
	**(N = 35)**
**Time between lung metastasis diagnosis and SBRT (month)**	
Median [Q1, Q3]	4 [2, 15]
**Pre-SBRT PSA (ng/mL)**	
Median [Q1, Q3]	1.7 [0.8, 3.0]
**Number of lung metastases treated with SBRT per patient**	
1	27 (77 %)
2	7 (20 %)
3	1 (3 %)
**ADT initiated before SBRT**	
Yes	17 (49 %)
No	18 (51 %)
**Duration of ADT received before SBRT (month)**	
Median [Q1, Q3]	16 [4, 47]
**Castration Status at SBRT**	
mCRPC	5 (14 %)
mCSPC	30 (86 %)
**PTV Volume (cc)**	
Median [Q1, Q3]	21.5 [11.3, 37.4]
**EQD2Gy_3_ (Gy)**	
Median [Q1, Q3]	130 [86.0, 144]

*Footnote: EQD2Gy_3:_ Equivalent dose in fractions of 2 Gy with a 3 Gy α/β. mCRPC: Metastatic Castration Resistant Prostate Cancer; mCSPC: Metastatic Castration Sensitive Prostate Cancer*.

### Oncological outcomes

**Fig. 1:**
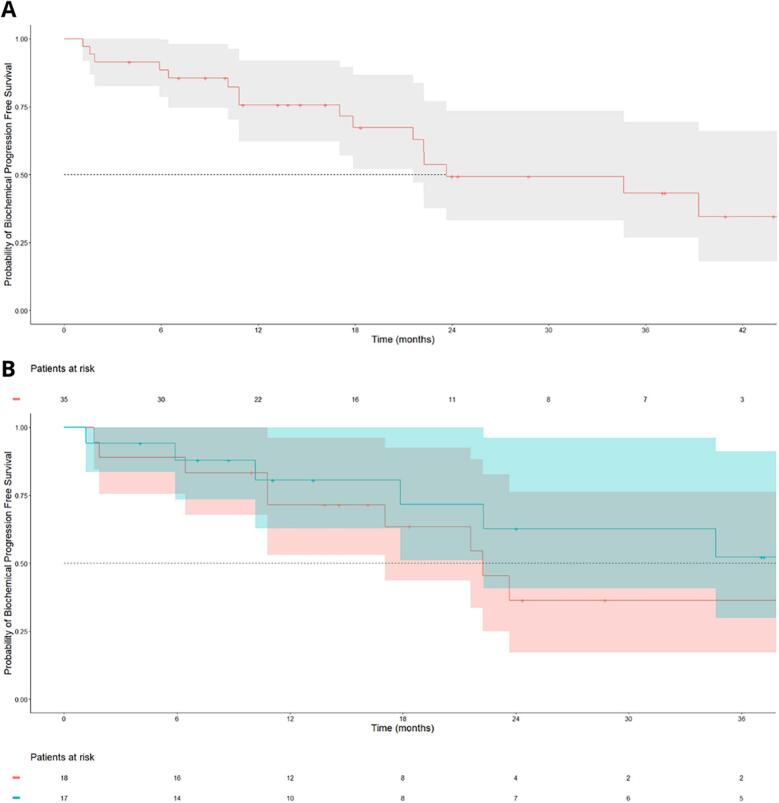
Progression free-survival (A: All patients; B: Patients with (blue) or without (red) ADT during SBRT). (For interpretation of the references to colour in this figure legend, the reader is referred to the web version of this article.).

After a median follow-up of 28.7 months [95 %CI:18.4;43.9], the median PFS was estimated at 21.6 months [95 %CI: 21.6; NA] (Fig. 1).

In the univariate and the multivariate analyses (Table 4), no variables were statistically significantly associated with PFS.

**Table 4:** Univariate and Multivariate Analysis for Progression Free-Survival.

	**HR (Univariate Model)**			**HR (Multivariate Model)**		
	**HR**	**IC95%**	**p-Value**	**HR**	**IC95%**	**p-Value**
**ISUP** (ref = 1–2–3)						
4–5	0.97	[0.33;2.86]	*0.960*			
**Pre-SBRT PSA**	1.04	[0.98;1.10]	*0.180*	1.06	[1.00;1.13]	*0.061*
**Number of lung metastases** (ref = 1)						
2––3	1.33	[0.47;3.76]	*0.585*			
**ADT initiated before SBRT** (ref = Yes)						
No	0.42	[0.14;1.24]	*0.117*	0.32	[0.10;1.05]	*0.059*
**Castration status at SBRT** (ref = CSPC)						
CRPC	0.24	[0.34;3.36]	*0.902*			
**PTV (cc)**	0.98	[0.96;1.01]	*0.207*			
**EQD2 Dose (Gy)**	1.01	[1.00;1.03]	*0.144*			

The median DMFS was 32.4 months [95 %CI: 22.2; NA] (Fig. S1). The median OS and LRFS were not reached (Figs. S2 and S3), due to the low number of events. At the date of last follow up, there were 2 (6 %) local recurrences with a 1-year and 2-year LRFS estimated at 100 % and 96.2 [95 %CI: 89.0;100] respectively.

**Fig. 2:**
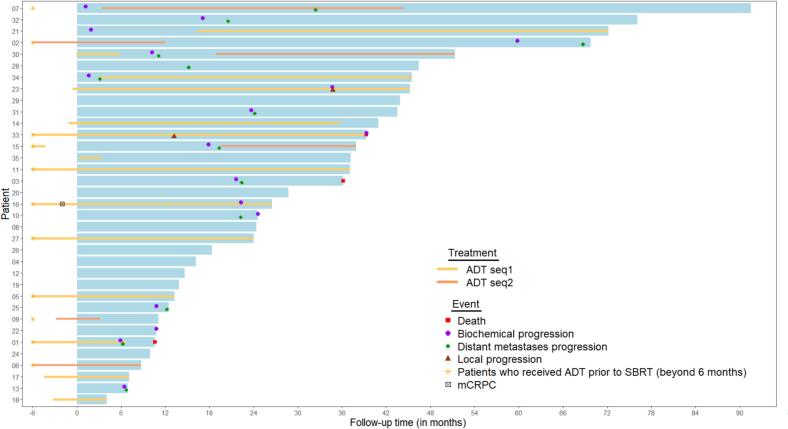
Individual patient outcomes.

More detailed information on the individual patient outcomes is available in the swimmerplot (Fig. 2).

### Safety

Defined by CTACE classification, 6 patients (17 %) developed grade 1 (n = 4, 11 %) or 2 (n = 2, 6 %) respiratory disorder. No Grade 3 or 4 toxicities were observed.

### Subgroup of patients treated with SBRT alone

There were 18 patients (51 %) with lung metastases treated with SBRT without any ADT given previously in the metastatic setting or concomitantly with SBRT. Clinical characteristics and SBRT details for this subpopulation are summarized in supplementary materials (Tables S1–S3). On this subpopulation, the 1-year PFS was estimated at 71.3 % [95 %CI: 53.1;96.2] and the 2-year at 36.3 % [95 %CI:17.2;76.3] (Fig. S4). The 1-year ADT free survival was estimated at 87.2 % [95 %CI: 71.9;100] and the 2-year at 77.5 % [95 %CI: 57.4;100] (Fig. S5).

## Discussion

To our knowledge, this is the first study conducted on oligometastatic prostate cancer with lung metastases treated with SBRT.

SBRT for PCa lung oligometastases demonstrated excellent local control, comparable to outcomes observed for bone and lymph node SBRT. Our results, with 1-year local control at 100 % and 2-year local control at 96.2.1 %, align closely with findings from three prospective studies evaluating the efficacy of stereotactic radiotherapy for bone or lymph node oligometastases. In the STOMP study, Ost et al. reported no local recurrences after a median follow-up of 3 years [15]. Similarly, in the ORIOLE study, Phillips et al. observed a local control rate of 98.9 % at 6 months, though no additional follow-up data were available [9]. Lastly, Siva et al. reported 1-year and 2-year local control rates of 97 % and 93 %, respectively [16].

SBRT for PCa lung oligometastases achieved biochemical control comparable to that reported for bone and lymph node SBRT in the ORIOLE and STOMP trials for patients who did not receive ADT. In the STOMP study, biochemical progression was defined as a PSA increase of ≥ 25 % and ≥ 2 ng/mL if baseline PSA was ≥ 2 ng/mL, or a PSA increase of ≥ 25 % if baseline PSA was < 2 ng/mL. In the ORIOLE study, progression was defined as a PSA increase of at least 2 ng/dL and 25 % above the nadir. Using a comparable definition of biochemical relapse in our study, our 12-month control rate of 71.3 % appears superior or at least comparable to the rates observed graphically in ORIOLE (approximately 70 % per Kaplan-Meier curves) and STOMP (approximately 45 % per Kaplan-Meier curves).

SBRT for PCa lung oligometastases seemed to lead to better ADT-free survival than that observed in the STOMP or Siva studies for lymph nodes or bone metastases. Comparisons with randomized studies should be made cautiously. Unlike prospective trials, where ADT is initiated based usually on the basis of specific criteria *(for example, in the STOMP study, ADT was initiated in cases of symptomatic progression, progression to more than three metastases or local progression of treated metastases),* in real-life practice, ADT initiation depends on physician habits and patient anxiety, and therefore is subject to variability. Nevertheless, this criterion is a pragmatic measure and enables us to assess the time saved by the patient without ADT in real life. This is significant, given the impact of ADT on patients' quality of life [17], physical function [18], sexual function [19] and cardiovascular risk [20,21]. In our study, ADT-free survival at 1 year was estimated at 87.2 % and at 2 years at 77.5 %, which seems to be better than the results observed graphically in STOMP with approximately 75 % at 1 year for patients treated with MDT on per-protocol analysis and those observed in Siva's study, in the subgroup of patients (n = 22) who had not started ADT prior to SBRT, with ADT-free survival at 2 years reported at 48 %. To maximize the duration without ADT, other strategies are currently being studied, including the addition of immunotherapy [22] or Lu177 PSMA radioligand therapy (NCT05560659) to SBRT, with pending results.

One of the reasons for the favorable results observed is the rigorous selection of patients. Most patients underwent metabolic imaging (PSMA or Choline PET/CT), which confirmed the oligometastatic nature of the disease [23]. Metabolic imaging also enabled the treatment of all metabolic lesions, which, as demonstrated by the ORIOLE study, resulted in significant improvements in progression-free survival (PFS) and distant metastasis-free survival.

However, our study has several limitations, inherent in its retrospective nature.

On the one hand, it involved a small number of patients with heterogeneous situations, which is easily explained by the rarity of the condition, despite the international collaborative effort.

On the other hand, follow-up after SBRT may seem short, but this can attributable to the recent and increasingly easy access to metabolic imaging, which enables these lung oligometastatic disease to be diagnosed more frequently. Stereotactic radiotherapy in these oligometastatic setting is also becoming more common, even outside clinical trials, encouraged by the publication of several promising prospective studies.

## Conclusion

SBRT for PCa lung oligometastases offers good oncological outcomes comparable to those reported for bone and/or lymph node metastases. Our results encourage the inclusion of patients with lung oligometastatic disease in clinical trials designed to assess the value of SBRT. In this respect, the results of the phase 3 (OLIGO-PRESTO, NCT04115007), enabling the inclusion of patients with lung oligometastases, will provide valuable insights.

## Author contributions

Study Design and Concept: Maximilien Rogé and Stéphane Supiot.

Acquisition of the Data: Maximilien Rogé, Patrick Bowden, Paul Conway, Ciro Franzese, Marta Scorsetti, Emmanuel Seront, Pierre Blanchard, Mario Terlizzi, Jonathan Khalifa, Corentin Pasquier, Ulriche Shick, Shankar Siva.

Analysis and interpretation of data: Maximilien Rogé, Julie Paul and Stéphane Supiot.

Drafting the manuscript: Maximilien Rogé and Stéphane Supiot.

Critical revision of the manuscript: Maximilien Rogé, Julie Paul, Patrick Bowden, Paul Conway, Ciro Franzese, Marta Scorsetti, Emmanuel Seront, Pierre Blanchard, Mario Terlizzi, Jonathan Khalifa, Corentin Pasquier, Ulriche Shick, Shankar Siva, Stéphane Supiot.

Statistical Analysis: Julie Paul.

Obtaining Funding: None.

Administrative, technical or material support: Laetitia Himpe, Clotilde Dorio, Cate Davey.

Supervision: Stéphane Supiot.

Other: Shankar Siva is supported by the Cancer Council Victoria Colebatch Fellowship.

## Data sharing statement

The data presented in this study are available upon request from the corresponding author. The data are not publicly available for confidentiality reasons.

## Funding information

This research received no external funding.

## Take home message

SBRT for PCa lung oligometastases offers good oncological outcomes comparable to those reported for bone and/or lymph node metastases.

## Declaration of competing interest

The authors declare that they have no known competing financial interests or personal relationships that could have appeared to influence the work reported in this paper.

## References

[1] Gravis G., Fizazi K., Joly F., Oudard S., Priou F., Esterni B. (2013). Androgen-deprivation therapy alone or with docetaxel in non-castrate metastatic prostate cancer (GETUG-AFU 15): a randomised, open-label, phase 3 trial. Lancet Oncol.

[2] Sweeney C.J., Chen Y.H., Carducci M., Liu G., Jarrard D.F., Eisenberger M. (2015). Chemohormonal therapy in metastatic hormone-sensitive prostate cancer. N Engl J Med.

[3] Fizazi K, Tran N, Fein L, Matsubara N, Rodriguez-Antolin A, Alekseev BY, et al. Abiraterone plus Prednisone in Metastatic, Castration-Sensitive Prostate Cancer. N Engl J Med. 27 juill 2017;377(4):352‑60. 10.1056/NEJMoa1704174 28578607

[4] Smith M.R., Hussain M., Saad F., Fizazi K., Sternberg C.N., Crawford E.D. (2022). Darolutamide and survival in metastatic, hormone-sensitive prostate cancer. N Engl J Med.

[5] Sweeney C.J., Martin A.J., Stockler M.R., Begbie S., Cheung L., Chi K.N. (2023). Testosterone suppression plus enzalutamide versus testosterone suppression plus standard antiandrogen therapy for metastatic hormone-sensitive prostate cancer (ENZAMET): an international, open-label, randomised, phase 3 trial. Lancet Oncol.

[6] Parker C.C., James N.D., Brawley C.D., Clarke N.W., Hoyle A.P., Ali A. (2018). Radiotherapy to the primary tumour for newly diagnosed, metastatic prostate cancer (STAMPEDE): a randomised controlled phase 3 trial. The Lancet Déc.

[7] Bossi A., Foulon S., Maldonado X., Sargos P., MacDermott R., Kelly P. (Nov 2024). Efficacy and safety of prostate radiotherapy in de novo metastatic castration-sensitive prostate cancer (PEACE-1): a multicentre, open-label, randomised, phase 3 study with a 2 × 2 factorial design. Lancet.

[8] Decaestecker K., De Meerleer G., Ameye F., Fonteyne V., Lambert B., Joniau S. (2014). Surveillance or metastasis-directed Therapy for OligoMetastatic Prostate cancer recurrence (STOMP): study protocol for a randomized phase II trial. BMC Cancer Déc.

[9] Phillips R., Shi W.Y., Deek M., Radwan N., Lim S.J., Antonarakis E.S. (2020). Outcomes of observation vs stereotactic ablative radiation for oligometastatic prostate cancer: the ORIOLE phase 2 randomized clinical trial. JAMA Oncol.

[10] Tang C., Sherry A.D., Haymaker C., Bathala T., Liu S., Fellman B. (2023). Addition of metastasis-directed therapy to intermittent hormone therapy for oligometastatic prostate cancer: the EXTEND phase 2 randomized clinical trial. JAMA Oncol.

[11] Gandaglia G., Karakiewicz P.I., Briganti A., Passoni N.M., Schiffmann J., Trudeau V. (2015). Impact of the Site of Metastases on Survival in Patients with Metastatic Prostate Cancer. Eur Urol Août.

[12] Halabi S., Kelly W.K., Ma H., Zhou H., Solomon N.C., Fizazi K. (2016). Meta-analysis evaluating the impact of site of metastasis on overall survival in men with castration-resistant prostate cancer. J Clin Oncol.

[13] Budnik J., Suri J., Bates J.E., Bylund K.C., Milano M.T. (2019). Prognostic significance of sites of visceral metastatic disease in prostate cancer: a population-based study of 12,180 patients. Clin Genitourin Cancer.

[14] Tappero S., Piccinelli M.L., Incesu R.B., Cano Garcia C., Barletta F., Morra S. (2024). Overall Survival of Metastatic Prostate Cancer Patients According to Location of Visceral Metastatic Sites. Clin Genitourin Cancer Avr.

[15] Ost P., Reynders D., Decaestecker K., Fonteyne V., Lumen N., De Bruycker A. (2018). Surveillance or metastasis-directed therapy for oligometastatic prostate cancer recurrence: a prospective, randomized, multicenter phase II trial. J Clin Oncol.

[16] Siva S., Bressel M., Murphy D.G., Shaw M., Chander S., Violet J. (Oct 2018). Stereotactic Abative Body Radiotherapy (SABR) for Oligometastatic Prostate Cancer: A Prospective Clinical Trial. Eur Urol.

[17] Nishiyama T., Kanazawa S., Watanabe R., Terunuma M., Takahashi K. (2004). Influence of hot flashes on quality of life in patients with prostate cancer treated with androgen deprivation therapy. Int J Urol Sept.

[18] Dacal K., Sereika S.M., Greenspan S.L. (2006). Quality of Life in Prostate Cancer Patients Taking Androgen Deprivation Therapy. J Am Geriatr Soc Janv.

[19] Ng E., Woo H.H., Turner S., Leong E., Jackson M., Spry N. (2012). The Influence of Testosterone Suppression and Recovery on Sexual Function in Men With Prostate Cancer: Observations From a Prospective Study in Men Undergoing Intermittent Androgen Suppression. J Urol Juin.

[20] Carneiro A., Sasse A.D., Wagner A.A., Peixoto G., Kataguiri A., Neto A.S. (2015). Cardiovascular events associated with androgen deprivation therapy in patients with prostate cancer: a systematic review and meta-analysis. World J Urol Sept.

[21] Levine G.N., D’Amico A.V., Berger P., Clark P.E., Eckel R.H., Keating N.L. (2010). Androgen-deprivation therapy in prostate cancer and cardiovascular risk: a science advisory from the American Heart Association, American Cancer Society, and American Urological Association: endorsed by the American Society for Radiation Oncology. Circulation.

[22] Rogé M., Pointreau Y., Sargos P., Meyer E., Schick U., Hasbini A. (2023). Randomized phase II trial in prostate cancer with hormone-sensitive OligometaSTatic relapse: Combining stereotactic ablative radiotherapy and durvalumab (POSTCARD GETUG P13): Study protocol. Clin Transl Radiat Oncol Mai.

[23] Perera M., Papa N., Roberts M., Williams M., Udovicich C., Vela I. (2020). Gallium-68 Prostate-specific Membrane Antigen Positron Emission Tomography in Advanced Prostate Cancer—Updated Diagnostic Utility, Sensitivity, Specificity, and Distribution of Prostate-specific Membrane Antigen-avid Lesions: A Systematic Review and Meta-analysis. Eur Urol Avr.

