# Supplementary material for: Stereotactic body radiotherapy for lung oligometastatic prostate cancer: An international retrospective multicenter study
Source: Clin Transl Radiat Oncol. 2025 Mar 10;52:100944. doi: 10.1016/j.ctro.2025.100944 (PMC11953985; doi:10.1016/j.ctro.2025.100944)
Supplement: Supplementary Data 1 [file mmc1.docx]

**Supplementary Materials**

[Figure S1: Distant Metastases Free-Survival 2](#_Toc191307176)

[Figure S2: Overall Survival 3](#_Toc191307177)

[Figure S3: Local Recurrence Free-Survival 4](#_Toc191307178)

[Figure S4: Biochemical Progression Free-Survival for patients without ADT started before or during SBRT 5](#_Toc191307179)

[Figure S5: Androgen Deprivation Therapy Free-Survival for patients without ADT started before or during SBRT 6](#_Toc191307180)

[Table S1: Distribution of Patients Across Different Centers and Countries 7](#_Toc191307181)

[Table S2: Primary tumor characteristics for patients without ADT started before or during SBRT 7](#_Toc191307182)

[Table S3: Metastatic disease characteristics for patients without ADT started before or during SBRT 8](#_Toc191307183)

[Table S4: Stereotactic Body Radiotherapy Details for patients without ADT started before or during SBRT 8](#_Toc191307184)

# Figure S1: Distant Metastases Free-Survival


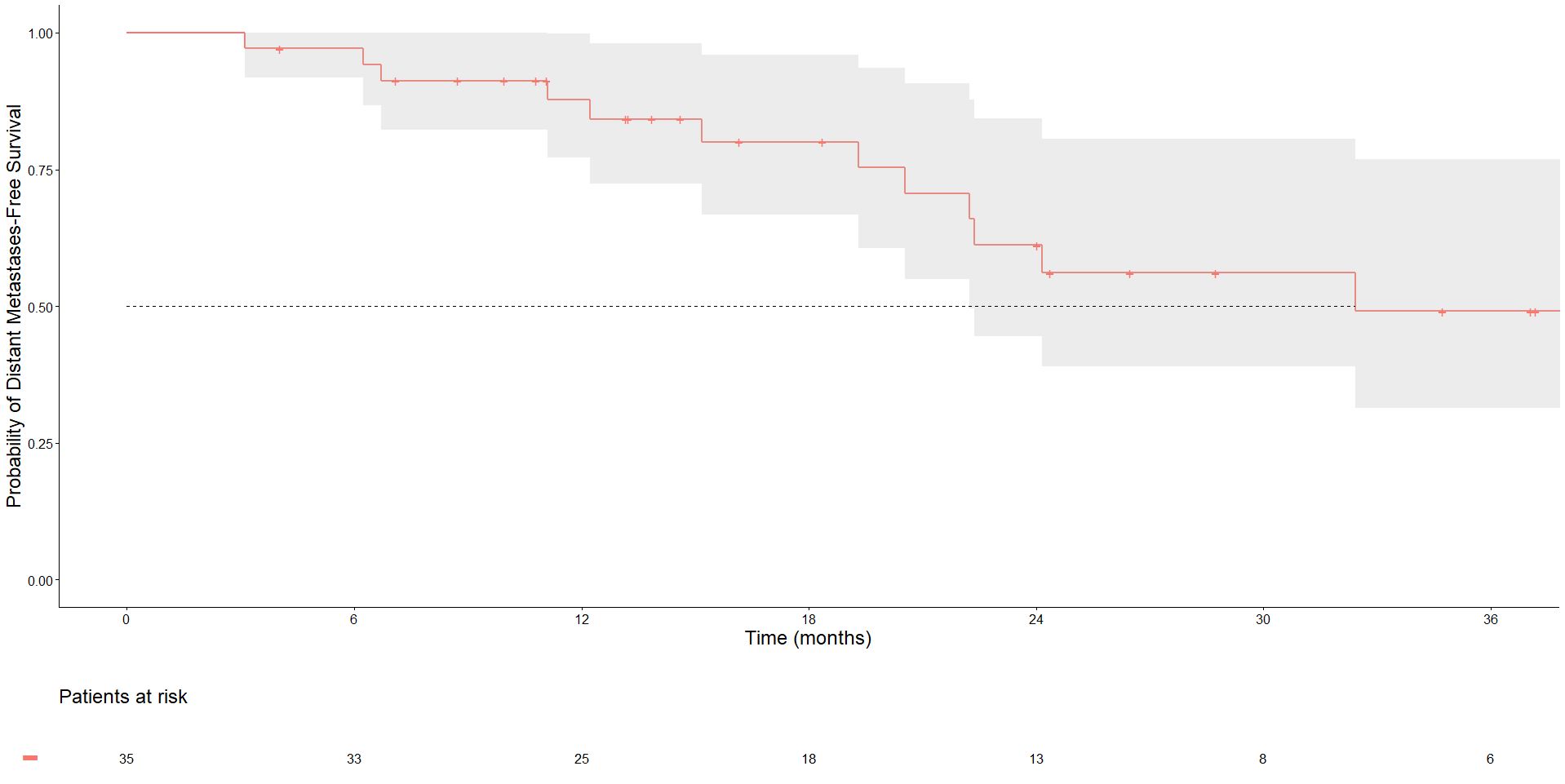


# Figure S2: Overall Survival


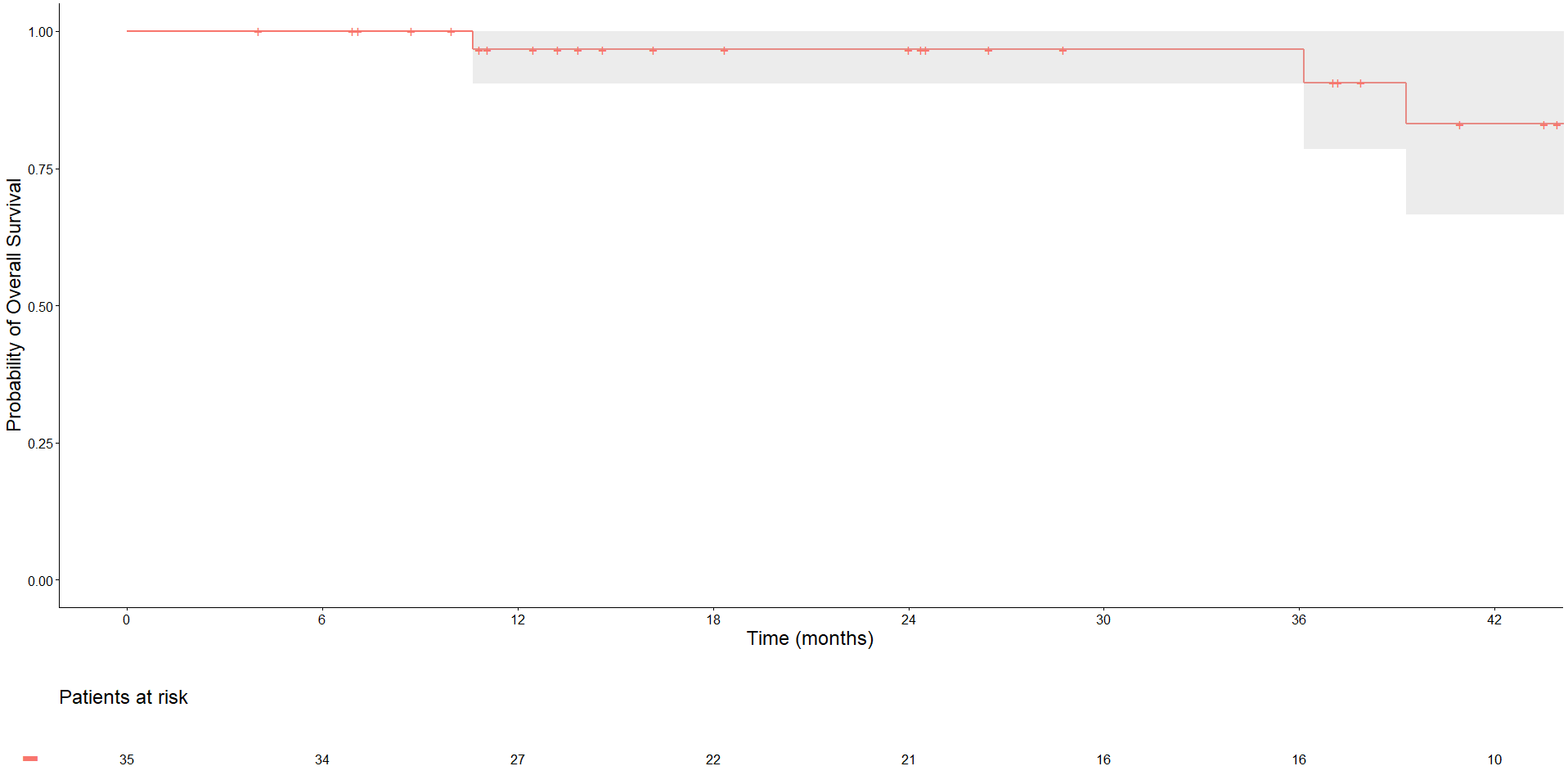


# Figure S3: Local Recurrence Free-Survival


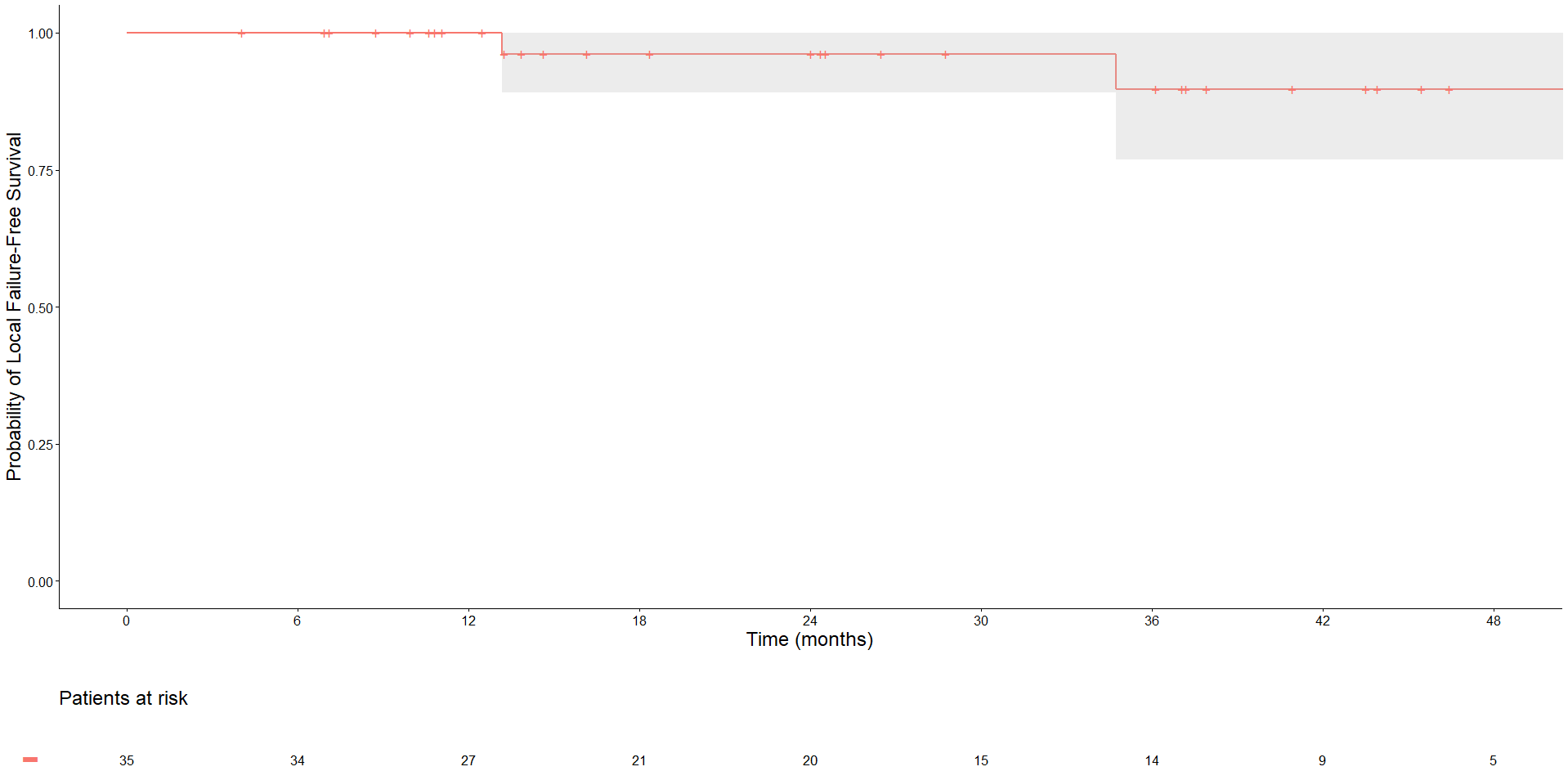


# Figure S4: Biochemical Progression Free-Survival for patients without ADT started before or during SBRT


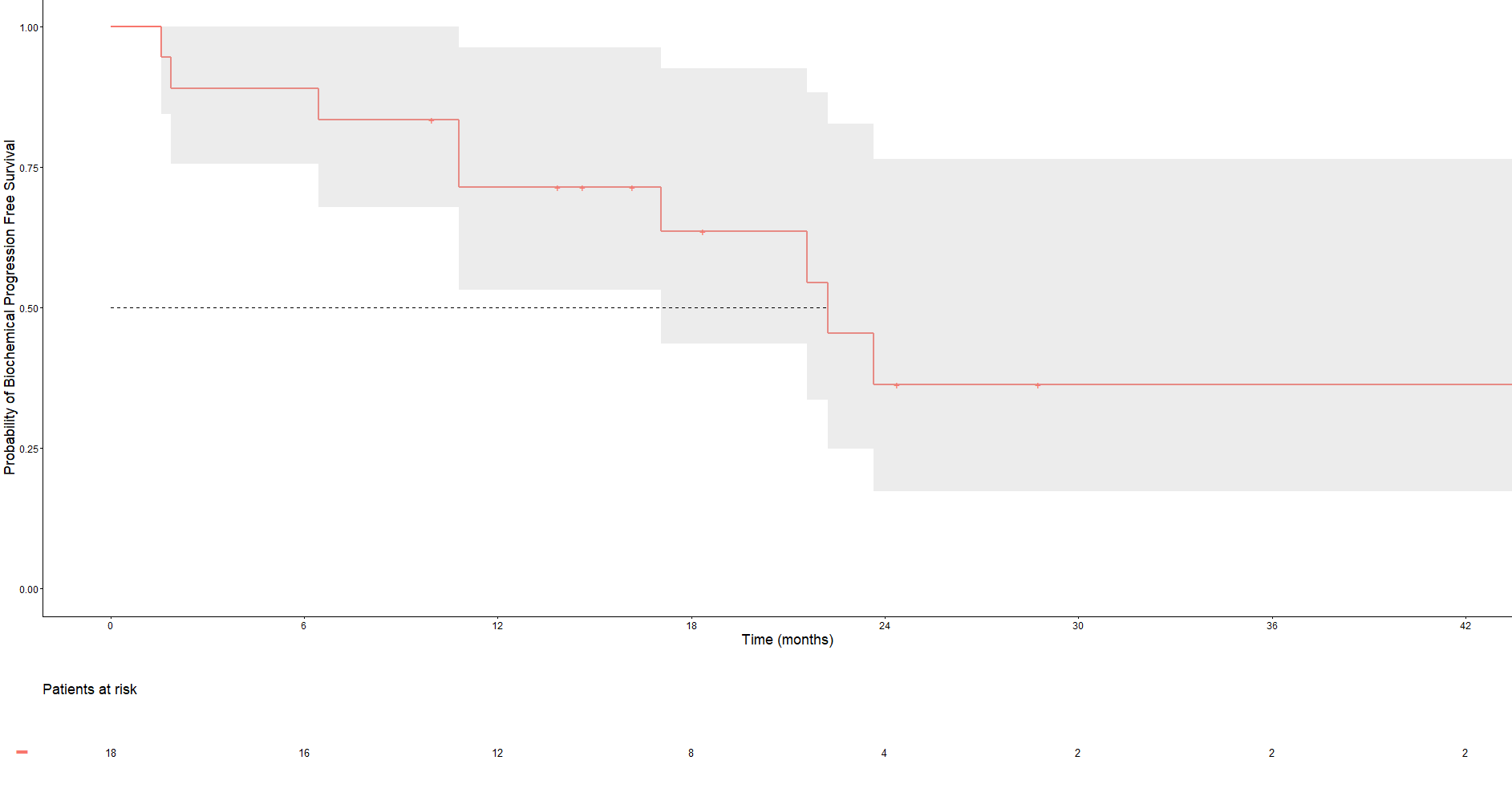


# Figure S5: Androgen Deprivation Therapy Free-Survival for patients without ADT started before or during SBRT


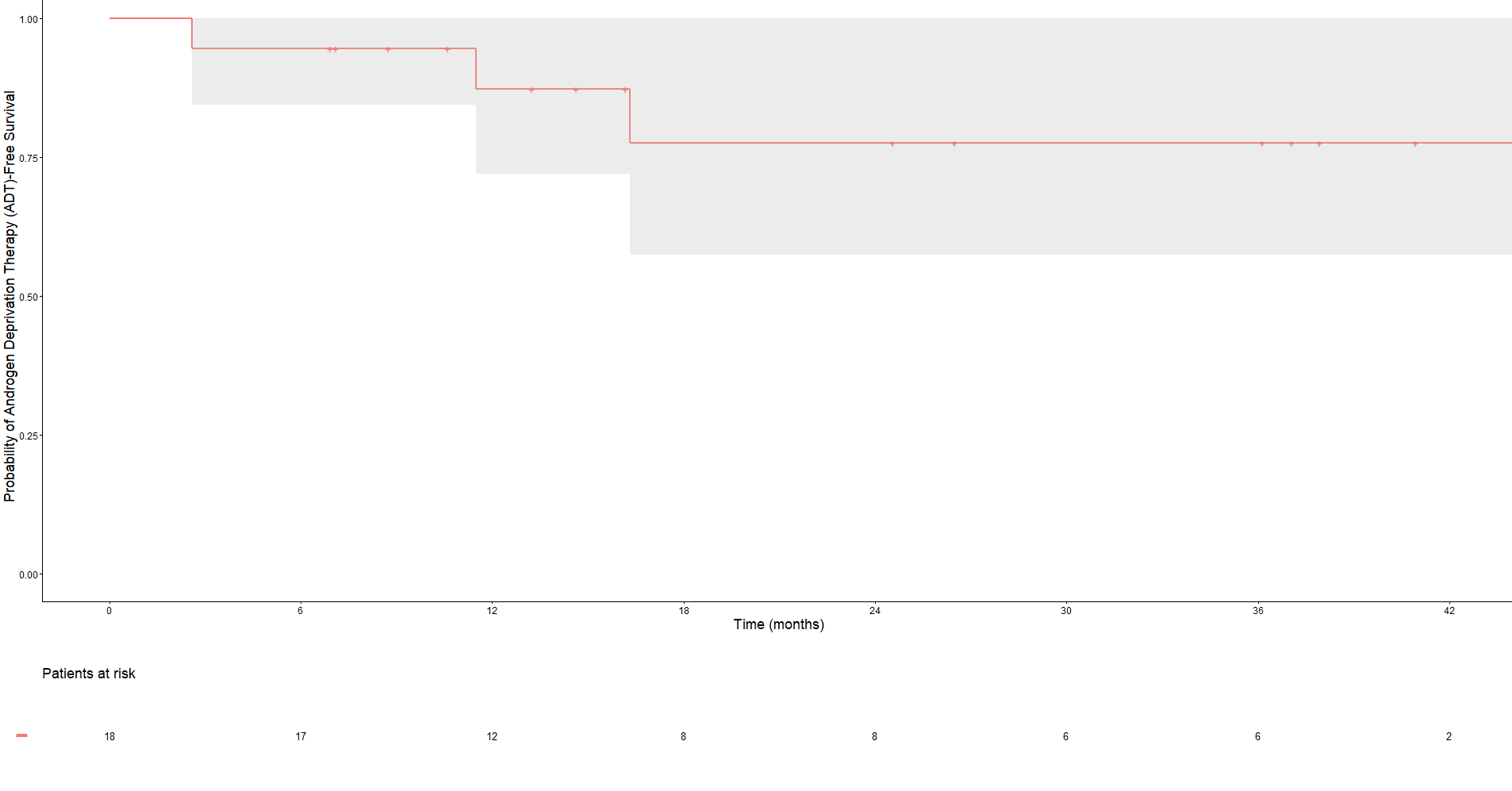


# Table S1: Distribution of Patients Across Different Centers and Countries

| Center | Total  (n=35) |
| --- | --- |
| Center n°1 | 9 |
| Center n°2 | 3 |
| Center n°3 | 2 |
| Center n°4 | 1 |
| Center n°5 | 10 |
| Center n°6 | 4 |
| Center n°7 | 6 |

# Table S2: Primary tumor characteristics for patients without ADT started before or during SBRT

|  | **Total**  **(N=18)** |
| --- | --- |
| **Age at PCa diagnosis (years)** |  |
| Median [Q1, Q3] | 65 [61, 71] |
| Missing | 0 |
| **Initial PSA (ng/mL)** |  |
| Median [Q1, Q3] | 8.9 [6.1, 12.5] |
| Missing | 4 |
| **ISUP** |  |
| 1 | 2 (11%) |
| 2 | 6 (35%) |
| 3 | 3 (18%) |
| 4 | 3 (18%) |
| 5 | 3 (18%) |
| Missing | 1 |
| **Prostate treatment** |  |
| Radical prostatectomy alone | 3 (17%) |
| Radical prostatectomy and Radiotherapy | 11 (61%) |
| Radiotherapy alone | 4 (22%) |
| No local treatment | 0 |

# Table S3: Metastatic disease characteristics for patients without ADT started before or during SBRT

|  | **Total** |
| --- | --- |
|  | **(N=18)** |
| **Number of metastases at first metastatic evolution** | |
| 1 | 11 (61%) |
| 2 | 6 (33%) |
| 3 | 1 (6%) |
| **Classification of Lung Oligometastatic Disease** | |
| Metachronous oligorecurrent disease | 17 (94%) |
| Synchronous oligometastatic disease | 1 (6%) |
| **Number of Lung metastases** | |
| 1 | 12 (67%) |
| 2 | 6 (33%) |
| **Metastatic imaging workup** | |
| PET Choline | 6 (33%) |
| PET PMSA | 12 (67%) |
| **Biopsy-proven lung metastasis** | |
| No | 11 (61%) |
| Yes | 7 (39%) |

# Table S4: Stereotactic Body Radiotherapy Details for patients without ADT started before or during SBRT

|  | **Total** |
| --- | --- |
|  | **(N=18)** |
| **Time between lung metastasis diagnosis and SBRT (month)** | |
| Median [Q1, Q3] | 2.7 [1.9, 3.8] |
| **Pre-SBRT PSA (ng/mL)** | |
| Median [Q1, Q3] | 1.8 [1.3, 2.9] |
| **Number of lung metastases treated by patient with SBRT** | |
| 1 | 14 (78%) |
| 2 | 4 (22%) |
| **PTV Volume (cc)** |  |
| Median [Q1, Q3] | 17.8 [11.4, 30.4] |
| **EQD2Gy_3_ (Gy)** | |
| Median [Q1, Q3] | 130 [80, 144] |
